# Nonmotorized recreation and motorized recreation in shrub‐steppe habitats affects behavior and reproduction of golden eagles (*Aquila chrysaetos*)

**DOI:** 10.1002/ece3.2540

**Published:** 2016-10-13

**Authors:** Robert J. Spaul, Julie A. Heath

**Affiliations:** ^1^ Department of Biological Sciences and Raptor Research CenterBoise State UniversityBoiseIDUSA

**Keywords:** human disturbance, nest attendance, nest survival, off‐road vehicles, protected species, trail management

## Abstract

Different forms of outdoor recreation have different spatiotemporal activity patterns that may have interactive or cumulative effects on wildlife through human disturbance, physical habitat change, or both. In western North America, shrub‐steppe habitats near urban areas are popular sites for motorized recreation and nonmotorized recreation and can provide important habitat for protected species, including golden eagles. Our objective was to determine whether recreation use (i.e., number of recreationists) or recreation features (e.g., trails or campsites) predicted golden eagle territory occupancy, egg‐laying, or the probability a breeding attempt resulted in ≥1 offspring (nest survival). We monitored egg‐laying, hatching and fledging success, eagle behavior, and recreation activity within 23 eagle territories near Boise, Idaho, USA. Territories with more off‐road vehicle (ORV) use were less likely to be occupied than territories with less ORV use (β = −1.6, 85% CI: −2.8 to −0.8). At occupied territories, early season pedestrian use (β = −1.6, 85% CI: −3.8 to −0.2) and other nonmotorized use (β = −3.6, 85% CI: −10.7 to −0.3) reduced the probability of egg‐laying. At territories where eagles laid eggs, short, interval‐specific peaks in ORV use were associated with decreased nest survival (β = −0.5, 85% CI: −0.8 to −0.2). Pedestrians, who often arrived near eagle nests via motorized vehicles, were associated with reduced nest attendance (β = −11.9, 85% CI: −19.2 to −4.5), an important predictor of nest survival. Multiple forms of recreation may have cumulative effects on local populations by reducing occupancy at otherwise suitable territories, decreasing breeding attempts, and causing nesting failure. Seasonal no‐stopping zones for motorized vehicles may be an alternative to trail closures for managing disturbance. This study demonstrates the importance of considering human disturbance across different parts of the annual cycle, particularly where multiple forms of recreation have varying spatiotemporal use patterns that create human–wildlife interactions.

## 1 Introduction

Recreation is increasing on public lands that provide important habitat for species of conservation concern (Balmford et al., 2015; Cordell, Green, & Betz, 2009). Interactions between recreationists and wildlife can result in human disturbance—the alteration of wildlife behavior (McGarigal, Anthony, & Issacs, 1991; Steidl, Kozie, Dodge, Pehovski, & Hogan, 1993) or physiology (Creel et al., 2002) from patterns that would occur without human influence (Frid and Dill 2002). Furthermore, impacts of recreation can negatively affect demographic rates (Watson, Bolton, & Monaghan, 2014) leading to decreased population abundance (French, González‐Suárez, Young, Durham, & Gerber, 2011) or avoidance of otherwise suitable habitat (Kangas, Luoto, Ihantola, Tomppo, & Siikamäki, 2010; Roche et al., 2016; Rodríquez‐Prieto & Fernández‐Juricic, 2005; Taylor & Knight, 2003). Also, recreation can affect wildlife via physical alteration of habitat quality or availability (Brehme, Tracey, McClenaghan, & Fisher, 2013; Shanley & Pyare, 2011) or changing trophic interactions (Geffroy, Samia, Bessa, & Blumstein, 2015). In some cases, local extinction of threatened species is possible (Losos, Hayes, Phillips, Wilcove, & Alkire, 1995; Newmark 1995, Ouren et al., 2007). Studies that simultaneously investigate the behavioral responses of individuals to different types of recreation and how these translate into population‐level outcomes may be particularly useful for identifying specific recreation–wildlife interactions that can be managed to reduce the negative effects of recreation on wildlife populations (Anthony, Steidl, & McGarigal, 1995; Beale & Monaghan, 2004; Kight & Swaddle, 2007; Liley & Sutherland, 2007; Rodríquez‐Prieto & Fernández‐Juricic, 2005).

As the volume of recreationists increases and types of recreation diversify (e.g., hiking, mountain biking, and motorcycle riding), multiple‐use management on public lands may become challenging when objectives to provide recreational opportunities for user groups may come into conflict with wildlife management objectives (Hobbs, Landry, & Perry, 2008). Studies of recreation–wildlife interactions have focused on either motorized (Buick & Paton, 1989; Harris, Nielson, Rinaldi, & Lohuis, 2014; McGowan & Simons, 2006) or nonmotorized recreation (Finney, Pearce‐Higgins, & Yalden, 2005; Reed & Merenlender, 2008) effects, and some study both (Brown et al., 2012; Costello, Cain, Nielson, Servheen, & Schwartz, 2013; González, Arroyo, Margalida, Sanchez, & Oria, 2006; McLeod, Guay, Taysom, Robinson, & Weston, 2013), but few study effects across several stages of the annual cycle of a species. Consideration of all forms of recreation across time is important because use by different types of recreationists is likely to vary seasonally and spatially, or humans may engage in more than one form of recreation in a visit. For example, a negative effect of motorized recreation could be the delivery of nonmotorized recreationists, such as walkers or runners, into remote areas that are farther away from parking lots or trailheads where recreationists congregate (Newsome, Moore, & Dowling, 2013). Spatiotemporal variation in type‐specific activity patterns could have cumulative or interacting effects that result in widespread and persistent disturbance of wildlife. Investigating type‐specific spatiotemporal patterns of recreationists and wildlife responses may help in identifying detrimental recreation–wildlife interactions during important phases, such as reproduction. Management strategies that vary over the course of the annual cycle can minimize impacts to wildlife during critical periods and allow for broader recreational use during other, less vulnerable, periods and reduce the conflict between managing for recreation and wildlife (Hammit, Cole, & Monz, 2015; Weston, Dodge, Bunce, Nimmo, & Miller, 2012).

In western North America, shrub‐steppe habitats near urban areas are popular sites for both nonmotorized recreation and motorized recreation and they can provide important habitat for protected species, including golden eagles (*Aquila chrysaetos*). Golden eagles are long‐lived, territorial raptors, with large home ranges, and limited suitable nesting locations (Kochert & Steenhof, 2002; Kochert, Steenhof, McIntyre, & Craig, 2002); thus, persistent disturbance within territories could have significant impacts on individuals and, if territories are abandoned, distributions (e.g., Fernández‐Juricic, 2000). Further, the golden eagle is a federally protected species in the United States under the Bald and Golden Eagle Protection Act, which prohibits any action that constitutes “take,” including disturbance, without appropriate mitigation (The Bald and Golden Eagle Protection Act (16 U.S.C. 668‐668c)). Understanding the underlying processes and demographic consequences of different types of eagle–recreationist interactions is therefore crucial for adaptive management that is designed to balance recreation opportunities and prevent disturbance to eagles.

Steenhof, Brown, and Kochert (2014) found that golden eagles in the Owyhee Front outside of Boise, Idaho, USA, had reduced productivity in ORV‐impacted areas compared to nonimpacted areas, during a period of rapid increase in ORV activity. However, Steenhof et al. (2014) suggested that further research was necessary to understand the underlying mechanisms by which ORVs may affect eagle productivity, in part because the metric of eagle productivity combined several aspects of eagle life history (territory occupancy, egg‐laying, and nest survival) and eagles at the study site were exposed to other forms of recreation. We investigated whether nonmotorized recreation (including horseback riding, mountain biking, and pedestrian uses such as hiking, walking, and running) and motorized recreation (including ORVs and road vehicles), affected eagle territory occupancy, egg‐laying, and nest survival, the probability a breeding attempt survived from egg‐laying to ≥1 offspring reaching fledging age (Steenhof & Newton, 2007). We hypothesized that human disturbance of eagles would depend on type‐specific temporal use patterns or spatial activity patterns, specifically, either trail density or proximity to recreation activity. We used images from motion‐activated trail cameras (Smallwood, Pollock, Wise, Hall, & Gaughan, 2012) to index use by recreation type at three different temporal scales: across the entire breeding season, during the early breeding season (from prebreeding to egg‐laying), and short‐term intervals within the breeding season (to represent intermittent recreation activity). In addition to monitoring occupancy and breeding outcomes, we observed eagle behavior, modeled which behaviors best predicted nest survival, and examined effects of recreation on behavior.

## 2 Methods

### 2.1 Study site

**Figure 1:**
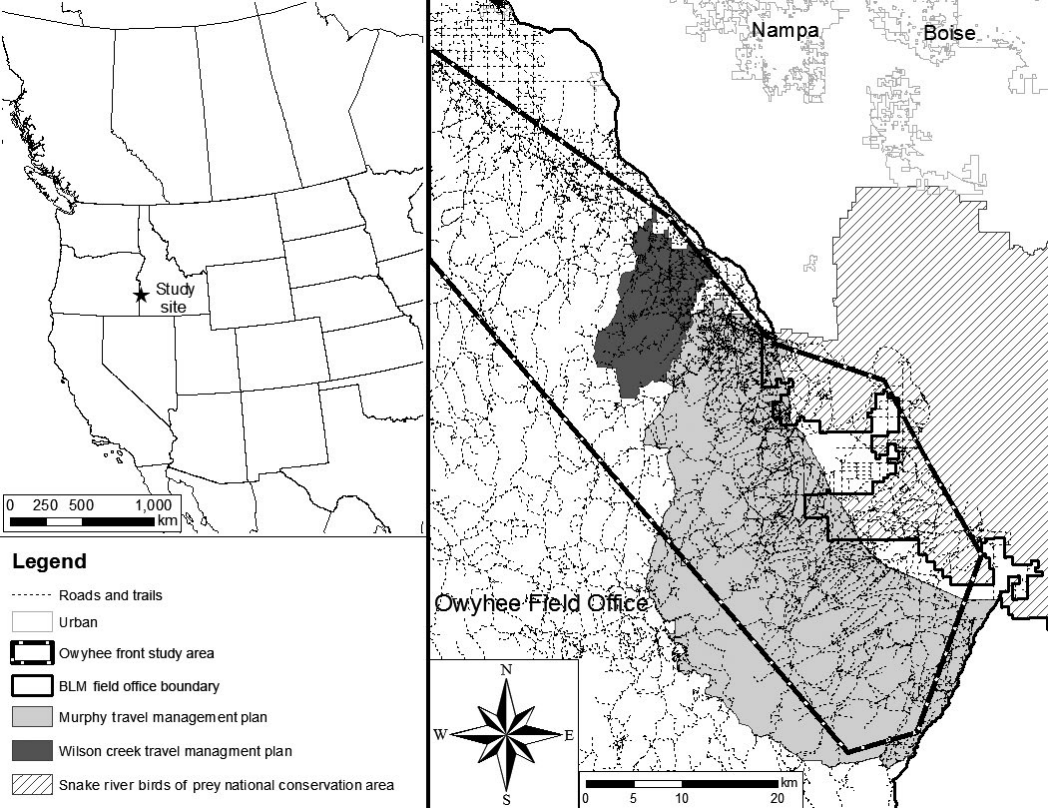
Owyhee Front, in southwestern Idaho. Golden eagle and recreation study site showing roads and trails and travel management areas in the study area.

Our study was conducted in southwestern Idaho, approximately 55 km from Boise (Figure 1). The study site is on public lands managed by the Bureau of Land Management (BLM), by the Owyhee Field Office (OFO), through multiple travel management plans (TMP), which define trail and road use and implement seasonal or permanent trail closures (Sutter, 2011, USDI, BLM, 2009). Study territories were within the Murphy TMP, the Wilson Creek TMP, the Morley Nelson Snake River Birds of Prey National Conservation Area, and other sites within the OFO, but outside designated travel management units (Figure 1). The area is a sagebrush (*Artemesia tridentata*)‐dominated shrub‐steppe ecosystem, including many canyons and rocky buttes, on the northern front of the Owyhee Mountains and south of the Snake River. The vegetative community is a mosaic of sagebrush subspecies, rabbitbrush (*Chrysothamnus* and *Ericameria* ssp.), antelope bitterbrush (*Purshia tridentata*), greasewood (*Sarcobatus* spp.), many other shrub species, and well‐established exotic annuals, principally cheat grass (*Bromus tectorum*).

### 2.2 Field techniques

We used a stratified‐random approach to select 23 golden eagle territories that varied in recreation use (based on personal observation and later verified with use estimates from trail cameras) and had nests that were visible from a distant observation point to minimize researcher disturbance. From mid‐January through mid‐April 2013 and 2014, we surveyed territories for adult eagles by checking the most recently used nests and alternate nests using protocols outlined in Pagel, Whittington, and Allen (2010) and Steenhof and Newton (2007). We considered territories occupied if we saw an incubating eagle, or a pair of eagles engaged in courtship behavior on more than two visits. We considered territories unoccupied if we detected no eagles after three, four‐hour observations, spaced approximately 30 days apart (Pagel et al., 2010). We surveyed all territories before eagles laid eggs. At occupied territories, we documented whether a pair laid eggs by the presence of an incubating eagle, the presence of eggs, eggshell fragments, or young in the nest. We made additional visits through early July to monitor nesting and conduct behavioral observations (see below). Laying dates were determined by backdating nestlings aged by sight (Hoechlin, 1976), or by the date halfway between the first confirmed evidence of incubation and the prior nest check. We considered nesting attempts successful if at least one nestling reached 51 days old and by the absence of dead nestlings within 200 m of the nest (Pagel et al., 2010; Steenhof & Newton, 2007). Fledging dates were estimated as the halfway point between nest checks when a ≥ 51‐day‐old nestling was in the nest and when fledging was confirmed.

Approximately every 30 days, from prebreeding (mid‐Jan) through fledging (6 July), we conducted four‐hour observations (n = 212) of potential nests or occupied nests from positions 600–1,200 m away to minimize researcher disturbance (Pagel et al., 2010). At least two observations occurred on both weekends and weekdays because recreation was higher on weekends than during weekdays (Appendix S1). Observers were either in a parked truck or pop‐up blind. We recorded the time that adult eagles were absent or their behavior every 5‐s. Behavior was categorized as the following: soaring, attacking, perched away from the nest (including preening), nest maintenance, copulation, incubating, brooding, perched at the nest (including preening and shading), feeding (actively feeding nestlings), and defensive posturing. If an eagle was flushed from the nest, behavioral surveys continued until the eagle returned to the nest and resumed its predisturbance activity. This protocol rarely resulted in the observation period extending by >30 min (~1% of observations). We identified males and females by size, copulatory behavior or by plumage or molt characteristics. Behavioral observations focused on the adult at the nest or the female if both eagles were present, but neither was at the nest, because females perform more parental care (Collopy, 1984). For analysis, behavioral categorizations were converted to percent time of the entire survey to standardize for survey duration. At territories where eagles laid eggs, behavioral observations of eagles lasted for an average of 4 hr (*SD*: 0.6 hr, n = 116), and occurred at 10 and 11 territories in 2013 and 2014, respectively.

While conducting behavioral observations of eagles, we identified and tallied all‐terrain vehicles (ATVs), rock crawlers, utility‐terrain vehicles (UTVs), dirt bikes, trucks, sport utility vehicles (SUVs), sedans, mountain bikes, horseback riders, and pedestrians within 1,200 m of nests. At territories where eagle pairs did not lay eggs, the most recently used nest was used as a spatial reference (hereafter called the “focal nest”). We calculated the number of recreationists per hour for each site and survey and used this value to predict behavior (see below).

We used multiple‐day camera‐based estimates of recreation use of trails near eagle nests for analysis of occupancy, egg‐laying, and nest survival. We sampled recreation use throughout each territory using trail cameras (Bushnell® HD Trophy Cameras and Moultrie® D55IR Gamespy Digital Cameras) placed along trails within 1,200 m of the focal nest. On some territories, there were several trails to select from. At these sites, we placed cameras on trails that were open, closest to the nest, and at points >100 m beyond the entrance or junction of a trail. Trail cameras were 8–10 m from trail edges, and sampled for five, eight‐ to 10‐day periods, every 5 weeks throughout the breeding season for each territory. Cameras were set to a 15‐s time delay between pictures. Although these recordings were likely to underestimate the total recreation use within a territory, the use estimates were positively correlated with counts of recreationists based on observation and considered a reliable index of use. An observer unfamiliar with each territory's location and reproductive outcome conducted image analysis by recording type of recreation activity, date, and time. We categorized recreationists into four groups: (1) ORVs (ATVs, UTVS, rock crawlers, and dirt bikes); (2) road vehicles (SUVs, trucks, and sedans); (3) nonmotorized riders (mountain bikes and horseback riders); and (4) pedestrians (recreationists traveling on foot). Multiple images of the same recreationist, distinguished by clothing or vehicle, was counted as a single event. Recreation use at each territory was calculated on a per day per trail basis, across three different timescales: (1) Breeding season recreation levels were represented by the averaged count per day per trail from 15 January to 6 July (Avg_Rec); (2) early season recreation levels were represented by the averaged count per day per trail from 15 Jan—mean annual laying date (PreLay_Rec); and (3) short‐term recreation use was the averaged count per day per trail within each interval between nest checks (Int_Rec).

We assessed proximity of each focal nest to a suite of recreation sites using trail and road data from the BLM‐OFO and imported into ArcGIS 10.1 (ESRI, Redlands, CA). We validated and corrected trails by digitizing from orthoimagery. We pooled all trail types for trail density (km/km^2^) calculations. We estimated trail density at three spatial scales, in fixed‐radius buffers of 400 m (50 ha), 1 km (314 ha) and 3 km (2,827 ha) from the focal nest. A 3‐km buffer around the nest was the median breeding season home‐range size of golden eagles in southwestern Idaho reported by Marzluff, Knick, Vekasy, Schueck, and Zarrielo (1997). Also, we measured the distances from focal nests to the nearest trail or road, the nearest open trail or road (as some trails in the study site were closed seasonally), the nearest campsite, the nearest recreational shooting spot, and the nearest trailhead (Table 1). Campsites were identified by the presence of fire rings or observation of camping. Recreational shooting sites were identified either by seeing people engaged in target practice or by finding large numbers of leftover shell casings. Nest–cliff height (the vertical distance between the nest and the bottom of the cliff) and nest–trail height (the vertical distance between the nest and the closest trail) were measured in the field using a clinometer and a laser rangefinder, after nestlings fledged or breeding attempts failed.

**Table 1:** Effect category, variable, description, and models that included the variables for recreation effects on territory occupancy (TO), egg‐laying (EL), and nest survival (NS) of golden eagles in the Owyhee Front, southwestern Idaho, 2013–2014

Effect category	Variable	Description	Included in models of
Disturbance via recreation: Timescale and type	Avg_ORV	Average of ORVs day^−1^, trail^−1^ during the eagle breeding season	TO, EL, and NS
	PreLay_ORV	Average of ORVs day^−1^, trail^−1^ before the mean laying date	TO, EL
	Int_ORV	Interval‐specific average of ORVs day^−1^, trail^−1^	NS
	Avg_Ped	Average of pedestrians day^−1^, trail^−1^ during the breeding season	TO, EL, and NS
	PreLay_Ped	Average of pedestrians day^−1^, trail^−1^ before the mean laying date	TO, EL
	Int_Ped	Interval‐specific average of pedestrians day^−1^, trail^−1^	NS
	Avg_Truck	Average of road vehicles day^−1^, trail^−1^ during the breeding season	TO, EL, and NS
	PreLay_Truck	Average of road vehicles day^−1^, trail^−1^ before the mean laying date	TO, EL
	Int_Truck	Interval‐specific average of road vehicles day^−1^, trail^−1^	NS
	Avg_Non_Motor	Average of horseback and Mt bikes day^−1^, trail^−1^ during the breeding season	TO, EL, and NS
	PreLay_Non_Motor	Average of horseback and Mt bikes day^−1^, trail^−1^ before mean laying date	TO, EL
	Int_Non_Motor	Interval‐specific average of horseback and mountain bikes day^−1^, trail^−1^	NS
Trail density	Trail_Density_3k	Trail density (km of trail/km^2^) at a 3 km buffer around the focal nest	TO, EL, and NS
	Trail_Density_1k	Trail density (km of trail/km^2^) at a 1 km buffer around the focal nest	TO, EL, and NS
	Trail_Density_400 m	Trail density (km of trail/km^2^) at a 400 m buffer around the focal nest	TO, EL, and NS
Proximity to recreation sites	Closest_Trail	Distance (m) to the closest trail or road	TO, EL, and NS
	Closest_Open_Trail	Distance (m) to the closest open trail or road	TO, EL, and NS
	Closest_Trail_Head	Distance (m) to the closest trail head	TO, EL, and NS
	Closest_Shoot	Distance (m) to the closest recreational shooting spot	TO, EL, and NS
	Closest_Camp	Distance (m) to the closest campsite	TO, EL, and NS
	Nest–trail height	Vertical distance (m) from the nest to the closest trail	NS
Nest characteristics	Year	Year of breeding attempt	NS
	Age	Number of days since estimated laying date	NS
	Middate	Middle Julian day of interval	NS
	Stage	Whether the pair is incubating or brooding	NS
	Nest–cliff height	Vertical distance (m) from the nest to the cliff bottom	NS

### 2.3 Statistical analysis

Trail camera recordings lasted an average of 9.4 days (*SD* = 2.0 days) and recreation was recorded an average of 47.2 days (*SD* = 6.9 days) per territory per season between 15 January and 6 July. We did not use images recorded on the first and last day of each survey so that all days would be full 24‐hr records. We used generalized linear mixed models (GLMMs) with a Poisson distribution and a log link to assess temporal variation in recreation use across the breeding season. Models included a random variable for territory identity. Trail camera survey data (n = 1,861) were categorized into weekdays (n = 1,359) and weekend days (n = 502) and then analyzed separately. We assessed both linear and polynomial models of Julian Week on predicting the use for each recreation type and identified the best explanatory models using Akaike's information criterion adjusted for small sample size (AICc) and a model selection approach (Burnham & Anderson, 2002), and assessed 85% confidence intervals on all parameters (Arnold, 2010, Appendix S1).

We created GLMMs with a binomial distribution and logit link to assess the influence of recreation use, proximity to recreation sites, and habitat features (Table 1) on naïve territory occupancy and whether eagle pairs at occupied territories laid eggs. Territory identity was included as a random variable in all models. We used naïve occupancy (not corrected for imperfect detection, MacKenzie et al., 2002) because eagles are highly detectable and there was no evidence to suggest that detection was affected by recreation thereby creating misleading trends (Brown, Steenhof, & Kochert, 2013). For the occupancy and egg‐laying analyses, we assessed the influence of recreation type and use, using an index of activity across the entire eagle breeding season (breeding season recreation levels) and recreation preceding the mean laying date (early season recreation levels). All numerical predictors were centered and scaled before analysis. We conducted pair‐wise Spearman correlation analyses for recreation use (at both temporal scales) and habitat features to check for multicollinearity in predictors. For any pair of variables with r > |.70|, we selected the variable with the most evidence for support (lowest AICc). We used a two‐stage process to evaluate factors that affect occupancy and egg‐laying. In the first stage, we used an exploratory approach by evaluating sets of single variable models within each of our hypotheses: disturbance (recreation type and use), trail density, and proximity to recreation features (listed as “Effect category” in Table 1). In the second stage, all possible combinations of variables within a hypothesis, with a ΔAICc < 2, were evaluated. We considered models with the lowest AICc and informative parameter estimates, specifically 85% confidence intervals that did not overlap 0 (Arnold, 2010), to be useful for inference.

We used nest survival models to evaluate the factors that affect whether or not a breeding attempt results in at least one fledging‐aged offspring, typically called nest success. Nest survival analyses allow for the modeling of temporally dynamic influences on nest success by estimating daily nest survival rates (DSR, Shaffer, 2004; Brown et al., 2013). We used logistic exposure nest survival models using the package nest survival, (courtesy M. Herzog) to assess the influence of recreation type and use, proximity to recreation sites, and habitat features, on nest survival of egg‐laying pairs. For this analysis, we used indices of recreation averaged across the season (breeding season recreation levels) to represent chronic disturbance patterns, and interval‐specific averages of recreation use within nest check intervals (short‐term recreation use) to represent intermittent disturbance patterns. In addition to the recreation covariates, we assessed the influence of year (2013 or 2014), nest age (0 = onset of incubation), chronology (represented by the date halfway between each nest check), nesting stage (incubating or brooding), and nest height on nest survival (Table 1). Because of the early and consistent nature of nest checks, nest survival models were applied from the estimated laying date, across a 43‐day incubation period (Kochert et al., 2002), through to the estimated fledging date. We used an information theoretic approach to evaluate nest survival models. Models with ΔAICc < 2 were considered to have the most support and variables with 85% confidence intervals that did not overlap zero were biologically informative. We calculated model‐averaged parameter estimates based on the models that made 100% of the weight in the hypothesis model comparison (Anderson, 2008).

We used pair‐wise Spearman correlation analyses to examine associations between the amounts of time eagles spent in each behavior or being absent from nest. We found that behaviors were highly correlated and generally grouped into two inversely associated categories of attending the nest or being absent. To avoid issues with multicollinearity, we evaluated single‐behavior models to determine which behavior best predicted nest survival and used the best behavioral predictor of nest survival as a response variable to evaluate recreation effects.

The percent of time spent at the nest (% At_Nest) was the best indicator of daily nest survival. The amount of time eagles spend at the nest varies with nest age (Collopy, 1984), so to remove the confounding effects of nest age, we used residuals from a general linear model of % At_Nest and nest age to represent age‐corrected percent of time at the nest. We used a linear mixed model to assess recreation type and use on age‐corrected % At_Nest. All linear models were made using functions (glmer and lmer) in the package lme4 (Bates et al., 2014), and analyses were performed in R 3.1.1 (R Core Team 2014). Descriptive statistics are reported as mean ± *SD*.

## 3 Results

Territory occupancy rates were 91.3% in 2013 and 86.9% in 2014. At occupied territories, 46.7% of 21 and 55% of 20 eagle pairs laid eggs in 2013 and 2014, respectively. Estimated mean laying dates were 6 March and 4 March, in 2013 and 2014, respectively. Mean nest–cliff height of egg‐laying pairs was 34.8 m ± 32.9 (range 8.9–152.3), and mean nest–trail height was 74.4 m ± 73.5 (range 20.4–209.6). Apparent nest success was 40.0% in 2013 and 36.4% in 2014. The number of fledglings per breeding pair (productivity) was 0.40 (n = 10) in 2013 and 0.45 (n = 11) in 2014.

**Figure 2:**
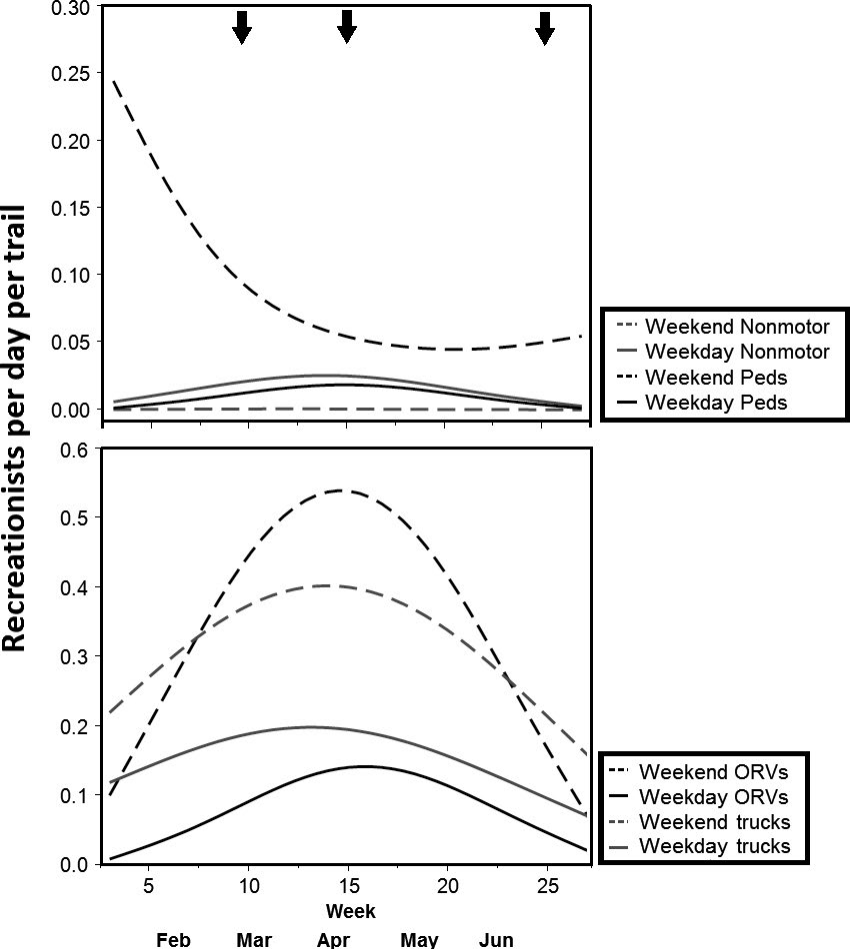
Breeding season trends in off‐road vehicles (ORVs), road vehicle (trucks), horseback and mountain bike riders (nonmotor), and pedestrian (peds) recreationists day^−1^, trail^−1^ across 23 golden eagle territories in the Owyhee Front, southwestern Idaho, in 2013–2014. Weekday (Monday–Friday) and weekend (Saturday and Sunday) use levels were modeled and displayed separately. Lines represent predicted values from generalized linear mixed models, with Julian Week and Julian Week^2^ as fixed effects and a random variable for territory identity. Vertical arrows across the top indicate the mean laying date, mean hatching date, and mean fledging date, respectively, from left to right. These figures show within‐season and across‐season variation in recreation use by different types of recreationists.

Breeding season recreation levels, across all territories, were 1.9 ± 5.1 (range 0–32.7) road vehicles per day per trail, 0.7 ± 1.0 (range 0–5.4) ORVs per day per trail, 0.5 ± 0.8 (range 0–3.77) pedestrians per day per trail, and 0.3 ± 0.5 (range 0–2.2) nonmotorized riders per day per trail based on data from trail cameras. Polynomial models of Julian Week, with a random variable for territory, were the best predictors of use for all recreation types, on both weekdays and weekends (Tables S1‐S8). Recreation activity was higher on weekends than on weekdays and changed over the course of the breeding season, for both weekdays and weekends (Figure 2). ORVs and road vehicles increased during the spring, peaked in the late spring, and then declined in the summer (Figure 2). Pedestrian activity was highest during late winter and decreased considerably as spring progressed (Figure 2). Nonmotorized riding activities occurred comparatively less frequently than other recreation types throughout the season, but peaked in the spring (Figure 2).

Trail density (km of trail/km^2^) within 400 m, 1 km, and 3 km of the focal nest was 2.2 ± 2.4 (range 0–7.7), 2.2 ± 1.8 (range 0.2–8.3), and 2.6 ± 1.7 (range 0.7–7.8), respectively. Mean distance to the closest trail was 307 m ± 257, mean distance to the closest open trail was 386 m ± 312, mean distance to the nearest trailhead was 2,471 m ± 1,731, mean distance to the nearest campsite was 2,314 m ± 1,554, and mean distance to the nearest shooting spot was 1,829 m ± 1,614.

**Figure 3:**
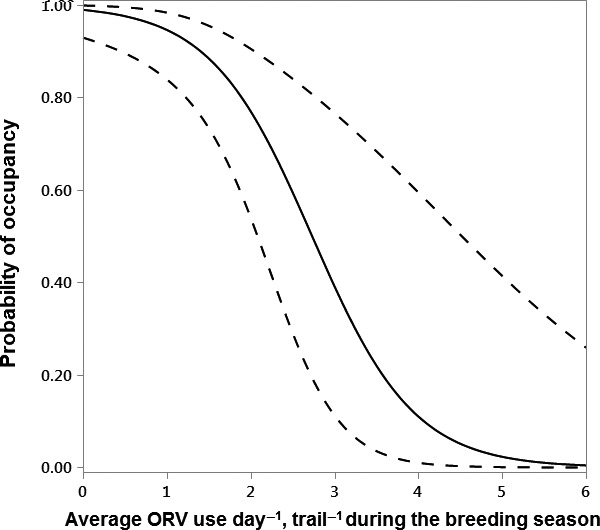
The relationship between average ORV use day^−1^, trail^−1^ during the breeding season (Avg_ORV), and golden eagle territory occupancy (n = 46), with solid line for model prediction, and dashed lines for 85% CIs. Territories with higher average ORV use during the breeding season were less likely to be occupied compared to territories with lower average ORV use during the breeding season.

ORV use averaged across the breeding season (Avg_ORV) was the best predictor of territory occupancy (Table 2). ORV use was negatively associated with territory occupancy (β = −1.6, CI = −2.8, −0.8, Figure 3) suggesting that the territories with the highest amount of ORV use were less likely to be occupied. There was some evidence that a model of trail density within 3 km of the focal nest predicted territory occupancy, but the confidence interval overlapped zero‐ and 3‐km trail density was correlated positively with Avg_ORV (r = 0.66); therefore, we did not create a model with both variables.

**Table 2:** AICc table showing candidate models, number of parameters (*K*), delta AICc (ΔAICc), cumulative weights (Cum.*w*_*i*_), parameter estimates (β), and lower and upper 85% confidence intervals for models used to explain the probability of golden eagle territory occupancy (n = 46) in southwestern Idaho, in 2013 and 2014. All models included the random variable for territory identity. See Table 1 for variable explanations

Model	*K*	ΔAICc	Cum.*w*_*i*_	β	Lower 85% CI	Upper 85% CI
Avg_ORV^a^	3	0.00	0.93	−1.6	−2.8	−0.8
Trail_Denisty_3k	3	5.55	0.99	−0.8	−1.6	−0.4
Closest_Trail	3	10.74	1.00	2.7	2.1	9.5
Closest_Shoot	3	11.48	1.00	2.5	0.4	8.3
Intercept‐only	2	12.45	1.00			

a AICc of top model = 21.74.

**Figure 4:**
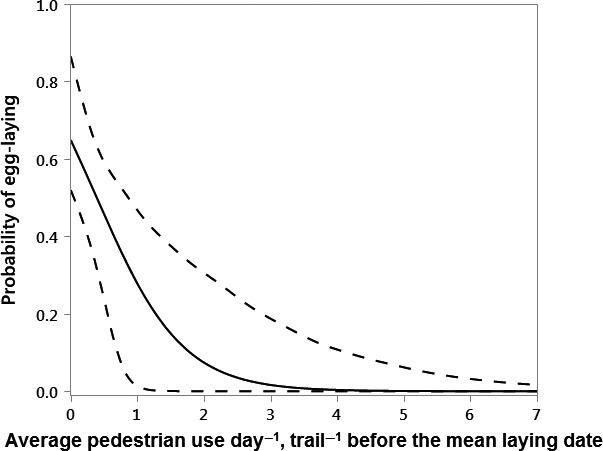
The relationship between average pedestrian use day^−1^, trail^−1^ before the mean laying date (PreLay_Ped), and the probability of a golden eagle pair laying eggs at occupied territories (n = 41), with solid line for model prediction, and dashed lines for 85% CIs. The probability of egg‐laying was inversely related to early season pedestrian use.

Early season pedestrian use (PreLay_Ped*)* was the best predictor of whether a pair laid eggs (Table 3), and had a negative effect (β = −1.6, CI = −3.8, −0.2, Figure 4) on the probability of a pair laying eggs. In addition, there was some evidence that early season nonmotorized rider use (PreLay_Non_Motor) predicted egg‐laying, but this predictor variable was positively correlated (r = 0.81) with early season pedestrian use.

**Table 3:** AICc table showing candidate models, number of parameters (*K*), delta AICc (ΔAICc), cumulative weights (Cum.*w*_*i*_), parameter estimates (β), and lower and upper 85% confidence intervals for models used to explain the probability of a pair of golden eagles laying eggs in the 2013 and 2014 breeding seasons in southwestern Idaho (n = 41). All models included the random variable for territory identity. See Table 1 for variable explanations

Model	*K*	ΔAICc	Cum.*w*_*i*_	β	Lower 85% CI	Upper 85% CI
PreLay_Ped^a^	3	0	0.6	−1.6	−3.9	−0.3
PreLay_Non_Motor	3	1.57	0.88	−3.6	−10.7	−0.3
Intercept‐only	2	3.23	1			

a AICc of top model = 57.90.

**Figure 5:**
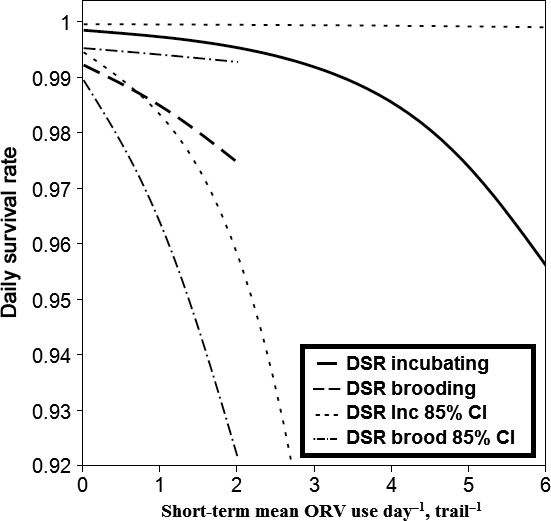
Daily nest survival rate (DSR) and short‐term mean ORV use day^−1^, trail^−1^ (Int_ORV) for incubating, and brooding golden eagles (n = 21) in the Owyhee Front, southwestern Idaho, in 2013–2014. Daily nest survival was higher during the incubation stage compared to the brooding stage, and daily nest survival declined with interval‐specific, short‐term ORV use, suggesting acute peaks in use may lead to nest failure.

Golden eagle nest survival was best explained by nest stage (model‐averaged β = 1.7, CI = 0.6, 2.8), and short‐term, interval‐specific ORV use (Int_ORV, Table 4). Int_ORV use was negatively associated with daily nest survival (model‐averaged β = −0.5, CI = −0.8, −0.2, Figure 5, Table S9), suggesting that short‐term peaks in ORV use may lead to nest failure of eagles. There was some evidence that the closest shooting spot (Closest_Shoot) and the closest campsite (Closest_Camp) influenced daily nest survival, but these variables were uninformative because their confidence intervals overlapped zero.

**Table 4:** AICc table showing candidate models, number of parameters (*K*), delta AICc (ΔAICc), and cumulative weights (Cum.*w*_*i*_) for models used to explain nest survival of golden eagle nests in the 2013 and 2014 breeding seasons in southwestern Idaho (n = 21). See Table 1 for variable explanations. See Table S9 for model‐averaged parameter estimates, standard errors, and 85% confidence intervals

Model	*K*	ΔAICc	Cum.*w*_*i*_
Stage + Int_ORV^a^	3	0	0.22
Closest_Shoot + Int_ORV + Stage	4	0.2	0.42
Closest_Camp + Int_ORV + Stage	4	0.47	0.59
Closest_Camp + Stage	3	1.22	0.71
Closest_Shoot + Stage	3	1.44	0.82
Stage	2	2.63	0.88
Int_ORV	2	4.36	0.9
Closest_Shoot	2	4.50	0.92
Closest_Shoot + Int_ORV	3	4.58	0.94
Closest_Camp	2	4.74	0.96
Closest_Camp + Int_ORV	3	4.90	0.98
Intercept‐only	1	4.92	1

a AICc of top model = 73.28.

**Table 5:** AICc table showing candidate models, number of parameters (*K*), delta AICc (ΔAICc),cumulative weights (Cum.*w*_*i*_), parameter estimates (β), and lower and upper 85% confidence intervals for models used to explain the influence of recreation covariates on age‐corrected nest attendance (n = 68 surveys). Recreationists per hour (hr^−1^) were estimated based on observations of recreation within 1,200 m of golden eagle nests. All models included the random variable for territory identity. For other variables, see Table 1 for explanations

Model	*K*	ΔAICc	Cum.*w*_*i*_	β	Lower 85% CI	Upper 85% CI
Pedestrians_ hr^−1^ a	4	0	0.55	−12.0	−19.2	−4.5
Intercept‐only	3	3.02	0.67			
Trail_Density_3k	4	5.01	0.71	−1.0	−3.6	1.7
All_Recreationists_ hr^−1^	4	5.02	0.76	−0.3	−1.0	0.5
Trail_Density_400 m	4	5.03	0.8	0.6	−1.2	2.5
ORVs_ hr^−1^	4	5.22	0.84	−0.1	−0.9	0.6
Trail_Density_1k	4	5.25	0.88	−0.3	−2.6	1.9
Nonmotorized riders_ hr^−1^	4	5.25	0.92	−1.7	−14.0	10.6
Road vehicles_hr^−1^	4	5.27	0.96	−0.4	−4.8	3.9
Closest_Open_Trail	4	5.28	1	0.0	0.0	0.0

a AICc of top model = 598.81.

**Figure 6:**
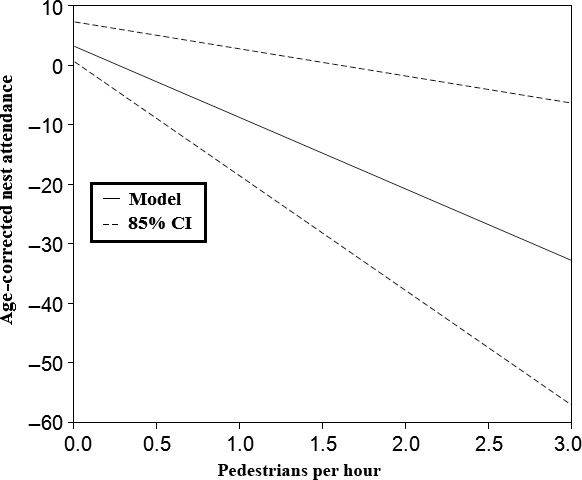
The relationship between age‐corrected nest attendance and pedestrians hr^−1^, who were observed within 1,200 m of golden eagle nests (n = 68 surveys) in the Owyhee Front, southwestern Idaho. Decreased nest attendance was associated with decreased daily nest survival.

Activity budgets of nesting golden eagles were typical for nesting semialtricial birds (Figure S1), and changed as expected throughout the stages of prebreeding, incubation, early brood‐rearing, and late brood‐rearing. Nest attendance was highest during incubation and decreased as nestlings aged. Behavior patterns were correlated with one another. For example, during prebreeding surveys, the percent of time perched at the nest correlated with nest maintenance (r = 0.70). During incubating surveys, the amount of time incubating was inversely correlated with the amount of time spent soaring (r = −0.84). During early brooding surveys, the amount of time spent brooding was negatively correlated with the amount of time an eagle was absent from the nest (r = −0.73). The total amount of time spent at the nest (% At_Nest) was a cumulative index of the nest attendance behaviors and was associated positively with nest survival. Age‐corrected % At_Nest was negatively associated with the number of pedestrians per hour (β = −11.99, CI: −19.25, −4.55, Figure 6), suggesting that as encounters with pedestrians increased, nest attendance decreased. Of the 50 pedestrians observed within 1,200 m of incubating or brood‐rearing eagles, most (66%) pedestrians initially reached the focal area from a truck or SUV, 30% initially arrived on an ORV, and 4% entered the area on foot.

## 4 Discussion

**Figure 7:**
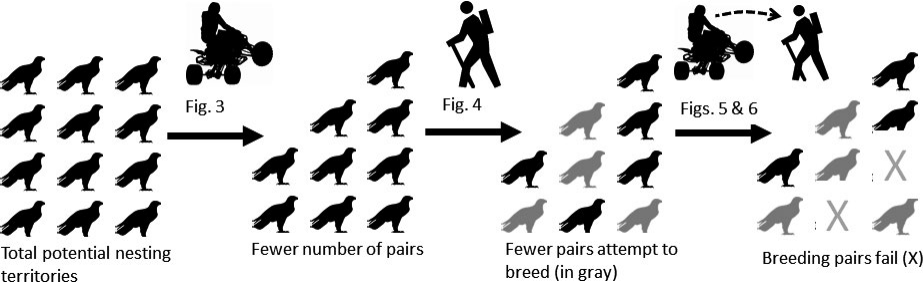
Representational figure of the cumulative effects of recreation on golden eagle reproduction in our study area. Potential pairs of eagles are represented by a single black eagle. From left to right, the number of occupied territories is lowered because of ORV use, early season pedestrian use is negatively associated with the probability of an eagle pair laying eggs, and, finally, nest survival is lower following ORV use peaks, that likely bring pedestrians near nests and pedestrians reduce adult nest attendance, leading to failure. Therefore, the actual number of successfully breeding pairs is lower than the potential number of successfully breeding pairs in the absence of recreation.

Golden eagle territory occupancy, egg‐laying, and nest survival were negatively associated with off‐road vehicle use, pedestrian and other nonmotorized recreation, and short‐term peaks in ORV use, respectively. These results suggest that, within our study site, multiple types of recreation influence specific stages of occupancy and reproduction. Combined, these have cumulative effects on golden eagles that could result in population‐level consequences through avoidance of otherwise suitable habitat, reduced egg‐laying, and increased nest failure (Figure 7). Further, adult nest attendance, a strong predictor of nest survival, was associated negatively with use by pedestrians who arrived on motorized vehicles. These results suggest that motorized vehicles may facilitate human disturbance events leading to nest failure by transporting recreationists who become pedestrians to areas near eagle nests. This illustrates the need to combine behavioral and reproductive monitoring for identifying the encounters and responses underlying disturbance events and effects on fitness. Finally, by assessing the effects of each form of recreation across different temporal scales (seasonal average, early season use, and short, interval‐specific peaks), we showed that uniformly high patterns of recreation and relatively short peaks in recreation can be detrimental to eagle occupancy and nest survival, respectively.

Territories with higher breeding season ORV use had the highest trail densities and were less likely to be occupied than territories with lower breeding season ORV use, despite low ORV use across all territories during the prebreeding period. Territory occupancy rates (91% in 2013, 87% in 2014) were similar to golden eagles in Alaska (mean = 86% from 1988 to 2010, McIntyre & Schmidt, 2012). Our results are consistent with golden eagle research from Finland, which showed reduced rates of occupancy in relation to tourist areas and greater length of snowmobile and ski trails (Kaisanlahti‐Jokimäki et al., 2008). Golden eagles in southwestern Idaho are typically year‐round residents, and there may be potential carry‐over effects associated with recreational use in fall and early winter, which this project did not assess. Alternatively, ORV activity also may be detrimental to the habitat that supports prey populations (jackrabbits, ground squirrels, upland game birds, etc.) of eagles. This effect on prey could occur through human disturbance of prey species or habitat degradation. Research on how recreation affects predator and prey interactions (e.g., Geffroy et al., 2015) would be useful for understanding why eagles were less likely to occupy territories with more ORV use.

Gill, Norris, and Sutherland (2001) suggested that life strategy options for disturbed wildlife depend on the availability of other suitable habitat. For territorial nonmigratory raptors that require specific sites for nest building, the availability of suitable nesting habitat is likely to be limited. Maintaining historical eagle nesting territories so that they are both available and have low risk factors for failure, to not become an ecological trap, is important. Like other cliff‐nesting raptors, nesting sites for golden eagles are limited and fewer suitable sites will result in a decrease in population size (Pauli, Spaul, & Heath, 2016; Watson & Whitfield, 2002). Behavioral observations at three adjacent, historically occupied territories, with high ORV volume and high trail density, suggested that one eagle pair used portions of all three nesting territories (R. Spaul, unpub. data). This behavior is consistent with other research showing that golden eagles may subsume adjoining territories when they become vacant (USGS, Snake River Field Station, unpub. data), perhaps in an attempt to compensate for compromised habitat quality by using larger home ranges (Andersen, Rongstad, & Mytton, 1990).

At occupied territories, visitation by pedestrians during the early portion of the breeding season negatively influenced the likelihood of golden eagles laying eggs, resulting in some territories being occupied by eagles that made no detectable breeding attempt. Adverse responses to pedestrians and nonmotorized riders before the mean laying date support the hypothesis that large raptors may be particularly vulnerable to disturbance at this crucial time (Watson, 2010). At this study site, the relatively high early season pedestrian use and comparatively low early season ORV use may lead to greater effects from pedestrian activity at this time of year. Pedestrian activities tend not to cause extensive habitat degradation, but the presence of humans may alter risk perception and result in a stress response that precludes eagles from laying eggs. Nonbreeding in periods of environmental stress may be a viable life history strategy for long‐lived organisms such as golden eagles that may maximize fitness through trade‐offs in current and future reproduction. For example, within a population, the proportion of eagle pairs that lay eggs can vary substantially (McIntyre & Adams, 1999; Steenhof, Kochert, & McDonald, 1997), but reduced probability of egg‐laying, year after year, may have detrimental effects on populations. The percentage of pairs laying eggs in this study (52.5%) was lower than average (70.0%) but within the observed range (38%–100%) of eagles in southwestern Idaho from 1971 to 1994 (Steenhof et al., 1997). The negative influence of pedestrian activity and nonmotorized riding on the probability of egg‐laying is consistent with results from golden eagles in Alaska, which show reduced reproductive potential near high pedestrian use (McIntyre & Schmidt, 2012). Similarly, Spanish imperial eagles (*Aquila adalberti*) had greater probability of flight reactions and flushed at greater distances in response to the unpredictable behaviors of nonmotorized recreationists, who tend to linger in an area longer than motorized recreationists (González et al., 2006). The same has been shown for waterbirds that flush at a farther distance for humans on foot than for cars (Guay et al. 2014, McLeod et al., 2013). Results from our project and these others provide evidence that management of recreation near golden eagle nest sites should consider the full suite of recreationists, not only motorized activity. Within our study site, seasonal trail closures apply only to motorized recreation activities (U.S. Department of the Interior, Bureau of Land Management, Murphy Subregion TMP. Environmental Assessment, 2009). Extending trail closures to pedestrian and perhaps other nonmotorized activities, especially during the early portion of the breeding season, could increase the number of pairs that lay eggs.

Nest survival was stage‐specific (lower during brood‐rearing than incubation) and negatively associated with short‐term peaks in ORV use (Figure 5). These findings support, and help explain, reduced productivity within areas of high ORV trail density, found by Steenhof et al. (2014). ORV use peaks from March to May and coincides with hatching and early brood‐rearing of nestling eagles (Figure 2). This is a time when nestling eagles are most susceptible to exposure if the parents are temporarily away from the nest (Watson, 2010). Additionally, nestlings are susceptible to starvation at this time, and ORV disturbance may prevent adequate provisioning by the parents, or a reduction of the prey base. It is also important to determine whether disturbance is causing eagles to flush from nests excessively, which may expose eggs and nestlings (Spaul & Heath, *in review*). Apparent nest success and productivity at this study site fell within typical ranges of some long‐term study sites (McIntyre & Schmidt, 2012; Steenhof et al., 1997), but both metrics are known to overestimate nest success (Shaffer, 2004).

Age‐corrected nest attendance of breeding eagles was a good predictor of nest survival. This result suggests that structured activity budgets can serve as an adequate measure of time necessary for successful breeding of golden eagles. Furthermore, age‐corrected nest attendance during the incubation and brood‐rearing stages were negatively associated with pedestrians that arrived within 1,200 m of the nest via ORVs (30%) or road vehicles (66%). This suggests the negative association between short‐term ORV use and nest survival may be the result of increased ORV‐based pedestrians. Animals may avoid pedestrians and other nonmotorized recreationists because their movements can be more varied and less predictable (Finney et al., 2005), and perceived as higher risk, than motorized recreationists who tend to make more predictable movements on trail networks at this site (Rob Spaul, unpub. data). Additionally, persecution from shooting continues to be a threat to golden eagles (Russel & Franson, 2014), and recreational shooting activities are common throughout this area. Continued threats from shooting may prevent habituation, or increase risk perception of recreationists on foot.

ORVs and trucks observed in this study rarely went off trail and often passed through an eagle territory within a few minutes. However, the canyons and cliffs on which eagles nest are landscape features of interest to recreationists, and eagle habitat may be an attractive spot for road vehicle and ORV users to disembark and begin hiking. This suggests that an area of overlap may exist between eagle nesting habitat and areas of high aesthetic value for recreationists, potentially leading to diminished habitat suitability (Braunisch, Patthey, & Arlettaz, 2011; Fernández‐Juricic, Sallent, Sanz, & Rodríguez‐Prieto, 2003). One management option may include implementation of “no‐stopping” zones, within close proximity to eagle nests. This could reduce the effective number of pedestrians in areas that are distant from trailheads or parking areas or do not typically have visitation from pedestrians arriving on foot. Further, recreationists may prefer, or comply with, no‐stopping zone regulations more often than trail closures; however, the efficacy of this strategy at decreasing disturbance to eagles would require further research.

Proximity of nests to recreation features (e.g., camping sites) was not associated with occupancy or reproductive rates. This suggests that the presence of trailheads, campsites, shooting spots, and trails does not deter eagles from occupying territories, laying eggs, or nesting successfully near these locations. Thus, if ORV, pedestrian and nonmotorized recreation use within 1,200 m was limited, recreation features outside of a 1,200‐m buffer could remain accessible to recreationists, without causing a change in eagle behavior. However, this study did not quantify or compare the size, or the relative usage of recreation features, which may have an influence on eagle reproduction. Other studies (Steenhof et al., 2014; Steidl et al., 1993) have found recreation features to be detrimental to productivity, and they still should be considered in management planning.

Nest–cliff height and the nest–trail height did not influence nest survival. This suggests that cliffs lying on lower rock outcrops, as they often do in this study site, are not less productive nesting sites than those lying on high cliffs or canyons. Furthermore, nesting sites that are vertically further from trails may be as susceptible to human disturbance as sites with less vertical separation.

The amount of pedestrian use was the largest negative influence on eagle nest attendance, but most pedestrians arrived near eagle nests via either an ORV or a road vehicle. An extensive network of roads and trails, extending throughout golden eagle habitat, brings people in contact with eagles that are disturbed by their presence. It remains to be seen whether enhanced recreation management can minimize loss in breeding potential. However, it is also important to reduce further expansion into remote areas, which are currently only marginally impacted by recreation. Many remote areas within this study site, and across the sagebrush‐steppe ecosystem, remain outside regulated travel management areas. Incorporating more eagle habitat into travel management areas and revising existing travel management regulations would both be important aspects of landscape‐scale golden eagle conservation.

## Conflict of Interest

None declared.
