# Supplementary material for: Nonmotorized recreation and motorized recreation in shrub‐steppe habitats affects behavior and reproduction of golden eagles (Aquila chrysaetos)
Source: Ecol Evol. 2016 Oct 13;6(22):8037–49. doi: 10.1002/ece3.2540 (PMC5108256; doi:10.1002/ece3.2540)
Supplement: Supplementary file 1 [file ECE3-6-8037-s001.docx]

**APPENDIX**

This appendix contains the statistical analysis and results for trail camera data that were used to assess temporal patterns of use by different forms of recreation in the Owyhee Front, southwestern Idaho. See the primary manuscript for methods used for camera placement. In addition, the appendix provides summaries of eagle behavior through four stages of the eagle breeding season: pre-breeding, incubation, early brood-rearing (0 - 21 day old nestlings), and late brood-rearing (22 - 71 day old nestlings; based on the oldest nestling, aged by sight (Hoechlin 1976)). See the primary manuscript for methods used for all behavioral surveys. The appendix also contains model averaged parameter estimates, standard errors, and 85% confidence intervals modeling the effects of recreation on nest survival. See the primary manuscript for methods used in nest survival modeling.

Statistical analysis- temporal patterns in recreation activity

Trail camera data: We downloaded all images from trail cameras and removed images from the first and last day of each survey so each sample only consisted of full 24-hr records. We used generalized linear mixed models (GLMMs) in R 3.1.1 (R Core Team 2014), using function “glmer” in package “lme4” (Bates et al. 2014), with a Poisson distribution, with territory as a random variable, to assess temporal patterns in recreation use across the entire breeding season, for each type of recreation. Trail camera survey days (n = 1861) were categorized into weekdays (n = 1359 trail camera days) and weekend days (n = 502 trail camera days), and then assigned a Julian Week to represent time of year. We created separate models for weekday and weekend recreation use. We assessed whether a linear model (Julian Week) or a polynomial model (Julian Week and Julian Week^2^) best predicted type-specific recreation use using AICc model selection (Burnham and Anderson 2002) and assessed 85% confidence intervals on all parameters (Arnold 2010).

Results

Polynomial models, with a random variable for territory were the best predictors of recreation use for all forms of recreations, on both weekdays and weekend days (Tables S.1-S.8). Model estimates of ORV, road vehicle, pedestrian and non-motorized use (Figure 2 in primary manuscript), are shown from 15 Jan to 15 Jul.

Activity budgets of golden eagles were typical for nesting raptors (Figures S.1), and changed predictably throughout the four stages (prebreeding, incubation, early brood-rearing, and late brood-rearing). Copulation (mean = 0.04% of time, SD = 0.01%) and nest maintenance (mean = 0.8%, SD = 1.9%) occurred during the prebreeding stage (n = 73 surveys), and eagles spent 1.5% (SD = 4.6%) of their time at the nest, though in many cases eagles were absent from the nest area for most of the survey period (mean = 61.5% of time, SD = 29.2%). Eagles spent 93.1% (SD = 10.3%) of their time incubating, and 0.7% (SD = 9.7%) perched at the nest or engaging in nest or egg maintenance (mean = 1.34%, SD = 1.74%) during the incubation stage (n = 26 surveys). Incubating eagles were only absent from the nest area 1.8% (SD = 6.0%) of the time.

Eagles spent 36.4% of their time brooding young during the early brood-rearing stage (n = 17 surveys), though this was highly variable (SD = 34.0%), due to variation in brooding time as nestlings matured. Adult eagles perched at the nest or shaded young for 26.6% of the time (SD = 23.7%), fed nestlings 8.4% of the time (SD = 7.1%), and performed nest maintenance 2.6% of the time (SD = 3.1%), spending a total of 74% of the time at the nest during the early brood-rearing stage. During the late brood-rearing stage (n = 25 surveys) eagles spent no time brooding during daytime surveys, but spent 13.3% (SD = 24.0%) of their time at the nest feeding (mean = 3.5%, SD = 5.8%), perching or shading (mean = 9.7%, SD = 19.5%), and maintaining the nest (mean = 0.2%). Eagles were completely absent from the nest area for much of the survey (mean = 47.2%, SD = 31.6%) at this time period, as nestlings became older.

**TABLES AND FIGURES**

**Table S.1.** AICc table showing the candidate models predicting ORVs per Weekend day per trail (n = 502). Top model: *ORVs_day* = -7.605 (± .576) + *Julian* *Week* * 0.499 (± 0.036) + *Julian* *Week^2^* * -3.180 (± 0.229). All models included the random variable of Territory.

**______________________________ ____________**

**Model**  **K** **ΔAICc** **Cum.*w_i_***

Julian Week + Julian Week^2^* 4 0.00 1.00

Julian Week^2^ 3 237.17 1.00

Intercept 2 239.38 1.00

Julian Week 3 240.53 1.00

*AICc of top model = 2079.98

**Table S.2.** AICc table showing the candidate models predicting ORVs per Weekday per trail (n = 1359). Top model: *ORVs_day* = -9.324 (± .828) + *Julian* *Week* * 0.486 (± 0.052) + *Julian Week^2^* * -2.860 (± 0.316). All models included the random variable of Territory.

**__________________ ________________________**

**Model**  **K** **ΔAICc** **Cum.*w_i_***

Julian Week + Julian Week^2^* 4 0.00 1.00

Julian Week 3 98.92 1.00

Intercept 2 105.92 1.00

Julian Week^2^ 3 106.90 1.00

*AICc of top model = 1900.76

**Table S.3.** AICc table showing the candidate models predicting Pedestrians per Weekend day per trail (n = 502). Top model: *PEDs_day* = 1.165 (± .661) + *Julian_Week* * -0.260 (± 0.036) + *Julian Week^2^* * 1.162 (± 0.262). All models included the random variable of Territory.

**__________________ ________________________**

**Model**  **K** **ΔAICc** **Cum.*w_i_***

Julian Week + Julian Week^2^* 4 0.00 1.00

Julian Week 3 16.57 1.00

Julian Week^2^ 3 48.12 1.00

Intercept 2 159.13 1.00

*AICc of top model = 1481.40

**Table S.4.** AICc table showing the candidate models predicting Pedestrians per Weekday per trail (n = 1359). Top model: *PEDs_day* = -11.627 (± 1.342) + *Julian* *Week* * 0.493 (± 0.079) +

*Julian Week^2^* * -3.090 (± 0.499). All models included the random variable of Territory.

**______________________________ ____________**

**Model**  **K** **ΔAICc** **Cum.*w_i_***

Julian Week + Julian Week^2^* 4 0.00 1.00

Intercept 3 46.54 1.00

Julian Week 2 46.54 1.00

Julian Week^2^ 3 28.54 1.00

*AICc of top model = 1232.13

**Table S.5.** AICc table showing the candidate models predicting Road Vehicles per Weekend day per trail (n = 502). Top model: *Rd_Veh_day* = -3.658 (± .447) + *Julian Week* * 0.209 (± 0.022) + *Julian Week^2^* * -1.397 (± 0.149). All models included the random variable of Territory.

**__________________ ________________________**

**Model**  **K** **ΔAICc** **Cum.*w_i_***

Julian Week + Julian Week^2^* 4 0.00 1.00

Intercept 2 92.12 1.00

Julian Week 3 92.23 1.00

Julian Week^2^ 3 93.53 1.00

*AICc of top model = 1934.55

**Table S.6.** AICc table showing the candidate models predicting Road Vehicles per Weekday per trail (n = 1359). Top model: *Rd_Veh_day* = -3.912 (± .442) + *Julian Week* * 0.160 (± 0.018) +

*Julian Week^2^* * -1.136 (± 0.116). All models included the random variable of Territory.

**__________________ ________________________**

**Model**  **K** **ΔAICc** **Cum.*w_i_***

Julian Week + Julian Week^2^* 4 0.00 1.00

Julian Week^2^ 3 87.17 1.00

Julian Week 3 99.34 1.00

Intercept 2 100.19 1.00

*AICc of top model = 3513.91

**Table S.7.** AICc table showing the candidate models predicting Non-Motorized riders per Weekend day per trail (n = 502). Top model: *Non_Motor_day* = -14.559 (± 1.794) + *Julian* *Week* * 0.499 (± 0.064) + *Julian* *Week^2^* * -3.466 (± 0.456). All models included the random variable of Territory.

**__________________ ________________________**

**Model**  **K** **ΔAICc** **Cum.*w_i_***

Julian Week + Julian Week^2^* 4 0.00 1.00

Julian Week^2^ 3 60.37 1.00

Julian Week 3 73.83 1.00

Intercept 2 80.77 1.00

*AICc of top model = 823.30

**Table S.8.** AICc table showing the candidate models predicting Non-Motorized riders per Weekday per trail (n = 1359). Top model: *Non_Motor_day* = -8.982 (± 1.262) + *Julian Week* * 0.339 (± 0.077) + *Julian Week^2^* * -2.270 (± 0.513). All models included the random variable of Territory.

**__________________ ________________________**

**Model**  **K** **ΔAICc** **Cum.*w_i_***

Julian Week + Julian Week^2^* 4 0.00 1.00

Intercept 2 19.80 1.00

Julian Week^2^  3 21.47 1.00

Julian Week 3 21.61 1.00

*AICc of top model = 898.88

**Table S.9.** Model averaged parameter estimates, model averaged standard errors (SE), and lower and upper 85% confidence intervals, for model of the effects of recreation on nest survival of golden eagles in the Owyhee Front, southwestern Idaho, in 2013 and 2014. Parameters listed make up 100% of model weight. See Table 1 in primary manuscript for complete variable descriptions.

| **Parameter** | **Estimate** | **SE** | **Lower 85% CI** | **Upper 85% CI** |
| --- | --- | --- | --- | --- |
| Stage | 1.708 | 0.804 | 0.550 | 2.867 |
| Int_ORV | -0.510 | 0.234 | -0.847 | -0.173 |
| Closest_Shoot | 0.530 | 0.384 | -0.023 | 1.083 |
| Closest_Camp | 0.526 | 0.389 | -0.034 | 1.086 |

**
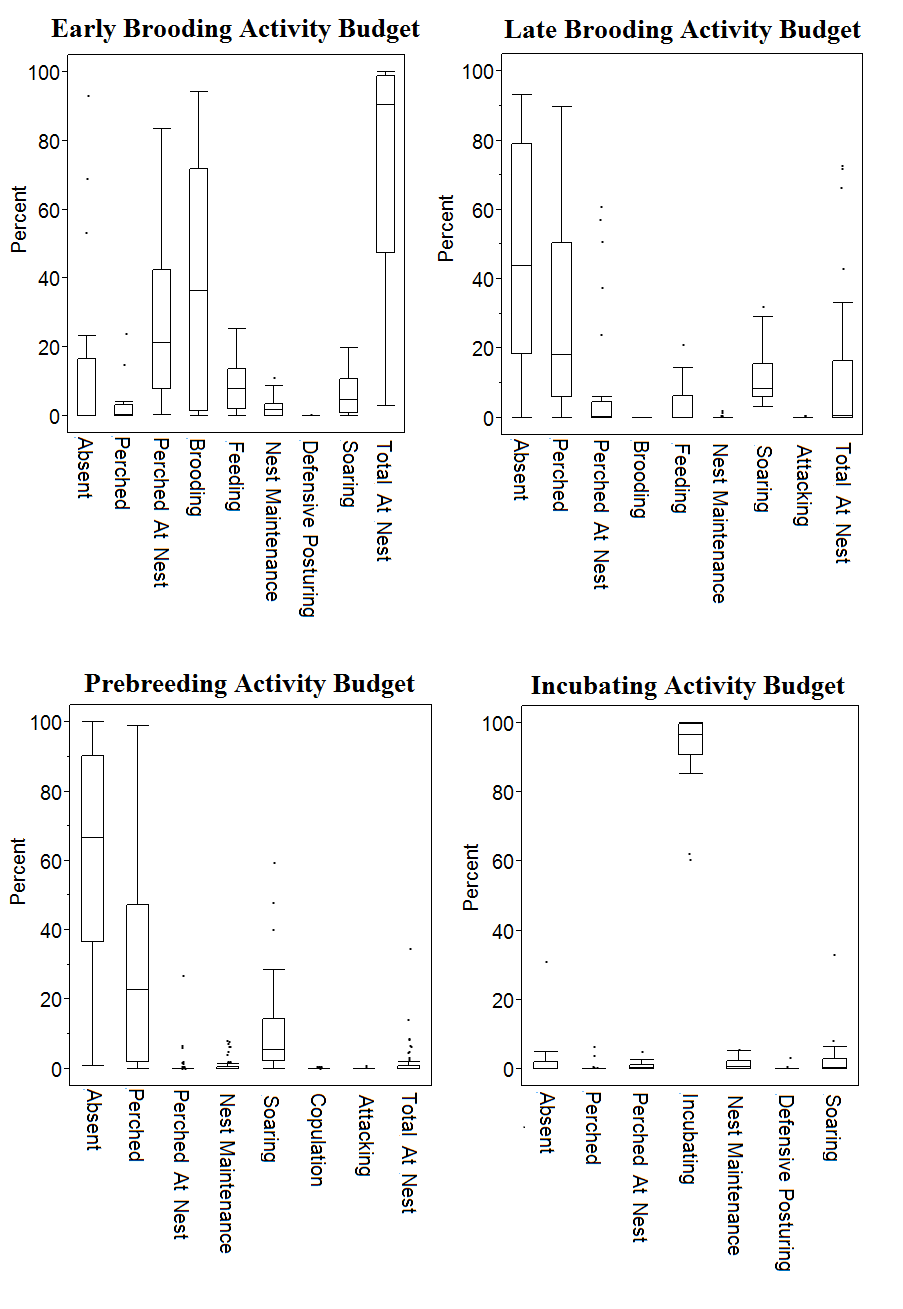
**

Figure S.1. Activity budgets of golden eagles at occupied territories during prebreeding, incubation, early brood rearing and late brood rearing stages. Behavioral surveys were categorized, to the following breeding stage categories: pre-breeding, incubation, early brood-rearing (0 - 21 day old nestlings), and late brood-rearing (22 - 71 day old nestlings; based on the oldest nestling, aged by sight (Hoechlin 1976)). Prebreeding surveys (n = 73) include all occupied territories. Incubation surveys (n = 26) include all occupied, egg-laying territories. Early (n = 17) and late brood rearing (n = 25) surveys include all territories where brood rearing occurred, and only include surveys from before nests fledged or failed.
